# Fitting the Pieces of the Puzzle Together: A Case of Median Arcuate Ligament Syndrome

**DOI:** 10.7759/cureus.18384

**Published:** 2021-09-29

**Authors:** Amit Sapra, Jacob Franke, Rachel Rahman, Christine E Albers, Priyanka Bhandari

**Affiliations:** 1 Department of Family and Community Medicine, Southern Illinois University School of Medicine, Springfield, USA

**Keywords:** postprandial abdominal pain, harjola-marable syndrome, dunbar syndrome, celiac artery compression syndrome, median arcuate ligament syndrome, chronic abdominal pain, vascular compression syndrome, celiac trunk

## Abstract

Median arcuate ligament syndrome (MALS), also known as Dunbar syndrome, is one of the many rare vascular compression syndromes attributed to celiac trunk compression by the median arcuate ligament of the diaphragm, with presentations ranging from completely asymptomatic to myriad gastrointestinal symptoms, including chronic abdominal pain (CAP), post-prandial pain, nausea and vomiting, anorexia, early satiety, and subsequently weight loss. We present a case of a 50-year-old female presenting with ongoing multiple chronic gastrointestinal symptoms, later attributed to the marked narrowing of her celiac axis secondary by the median arcuate ligament.

## Introduction

Celiac artery compression syndrome is one of the many vascular compression syndromes [1]. The syndrome poses challenges to the clinicians, with some patients being completely asymptomatic, whereas others can have many non-specific symptoms [1]. Despite its rarity, raising providers' awareness and comprehension about the underlying ischemic process being an external anatomic compression rather than an underlying embolic or an atherosclerotic process is paramount to understanding its surgical management.

## Case presentation

**Figure 1:**
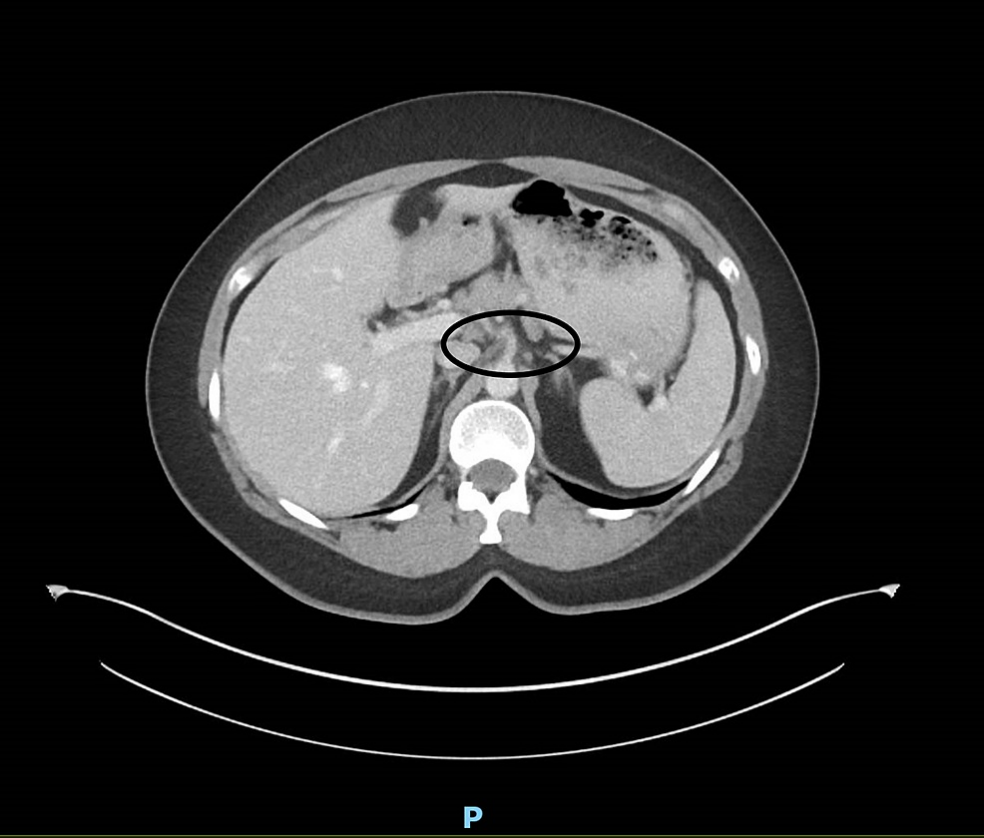
Celiac artery narrowing at its origin

**Figure 2:**
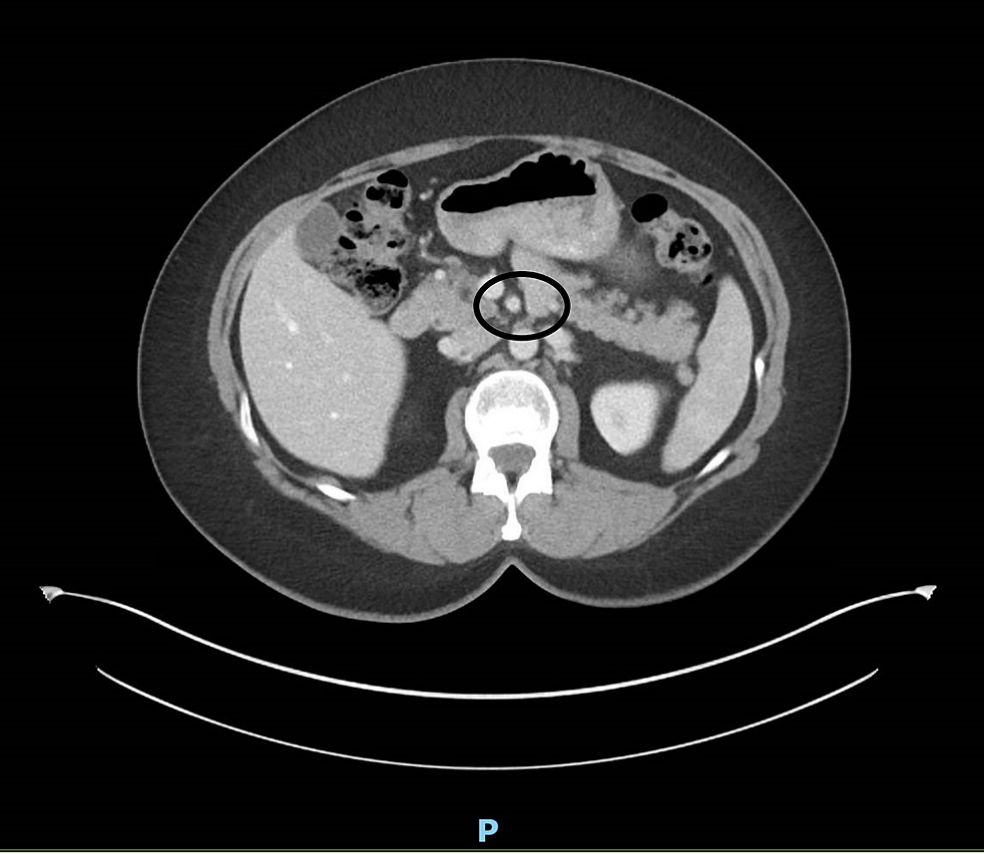
Superior mesenteric artery is patent

**Figure 3:**
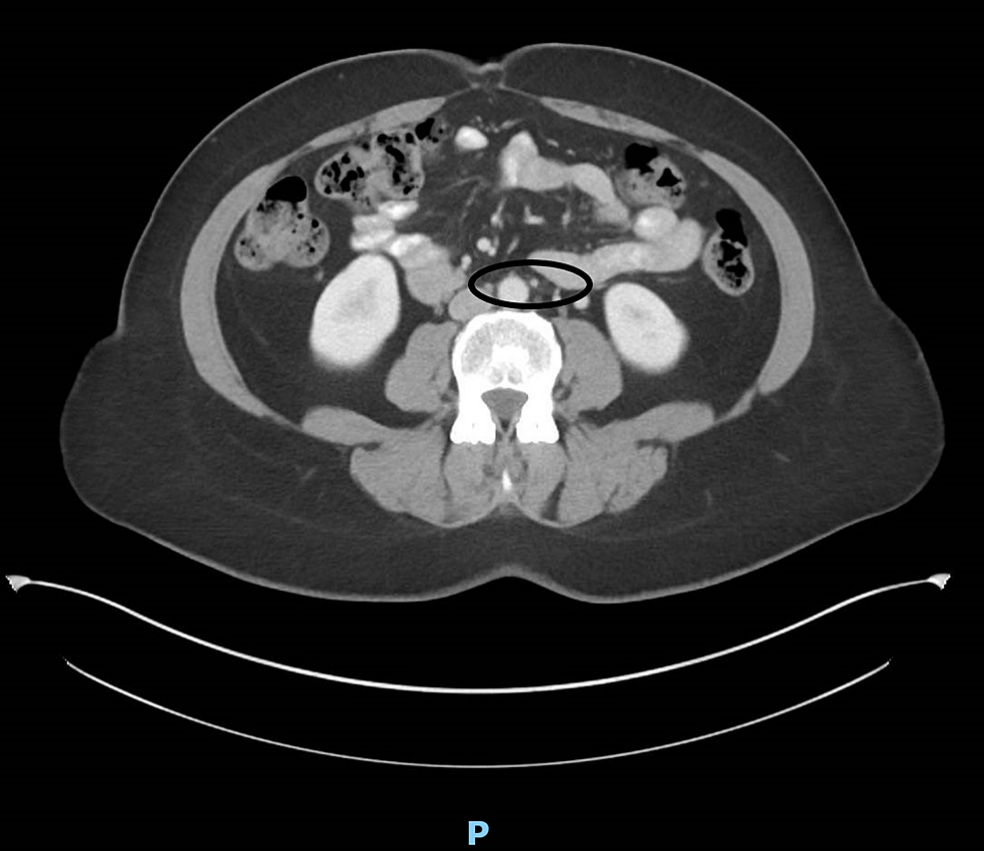
Inferior mesenteric artery is patent

A 50-year-old female with a past medical history significant for pericarditis had been presenting to her primary care provider with concerns about ongoing epigastric pain, worse with food ingestion, and intermittent radiation towards the back, worsening for the last few months. The pain was associated with nausea, vomiting, diarrhea, and early satiety. The severity of pain was moderate to severe and sometimes causing nocturnal awakenings. There was minimal response to a bland diet, the over-the-counter pain medications, and treatment with antacids, which she tried for almost three months. Physical examination was unremarkable. Her initial lab work including a complete blood count with differential kidney functions, liver functions, electrolytes, thyroid function tests, serum lipase, serum amylase, immunoglobulin E level, and urine culture and sensitivity were all reported as unremarkable. Subsequently, she underwent stool testing including hemoccult testing, stool white blood count, stool fecal fat, *Clostridium difficile* polymerase chain reaction, and calprotectin which were also reported unremarkable. The patient was referred to a gastroenterologist, who ordered a computerized tomography of her abdomen with contrast, which showed evidence of marked narrowing at the origin of the celiac artery, secondary to compression by the median arcuate ligament of the diaphragm (Figure 1). The scan reported soft-tissue stranding of the celiac artery, but the superior mesenteric and the inferior mesenteric artery were found to be patent (Figures 2, 3).

**Figure 4:**
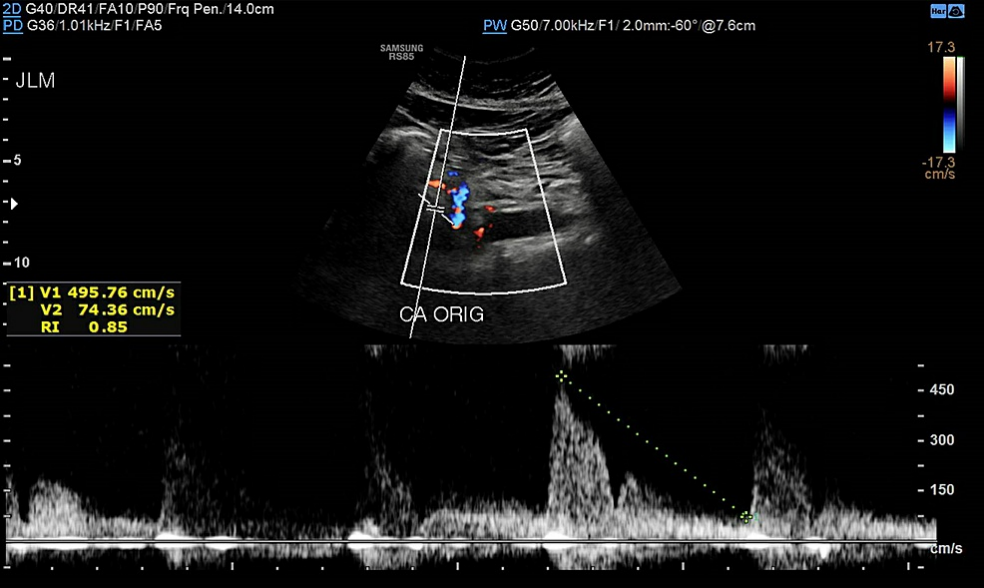
Celiac artery at the origin with a velocity measuring 495.76 cm/s

**Figure 5:**
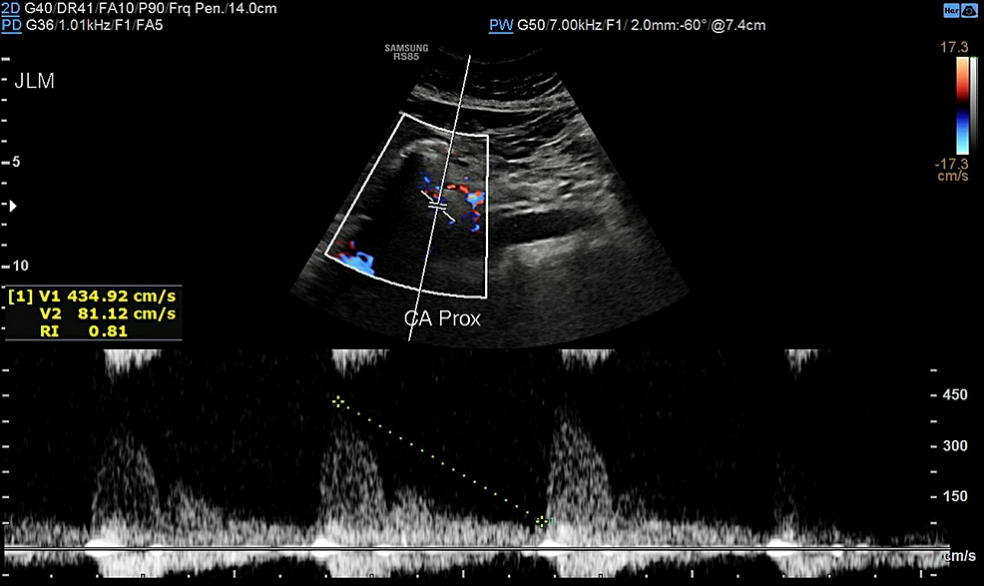
Celiac artery proximally with a velocity measuring 434.92 cm/s

**Figure 6:**
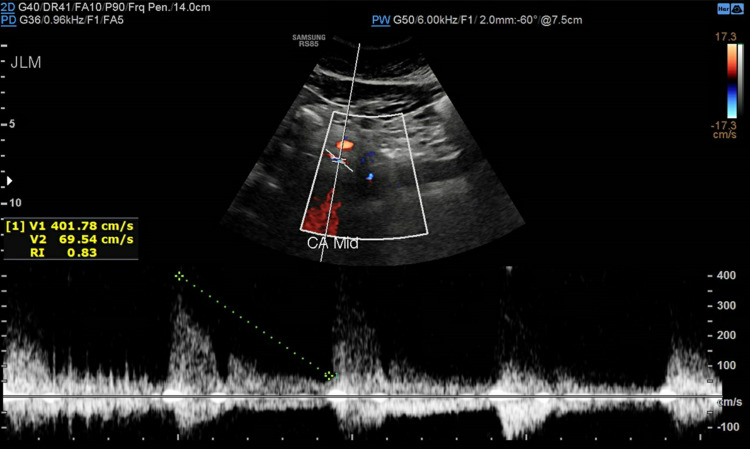
Celiac artery in the mid-portion with a velocity measuring 401.78 cm/s

**Figure 7:**
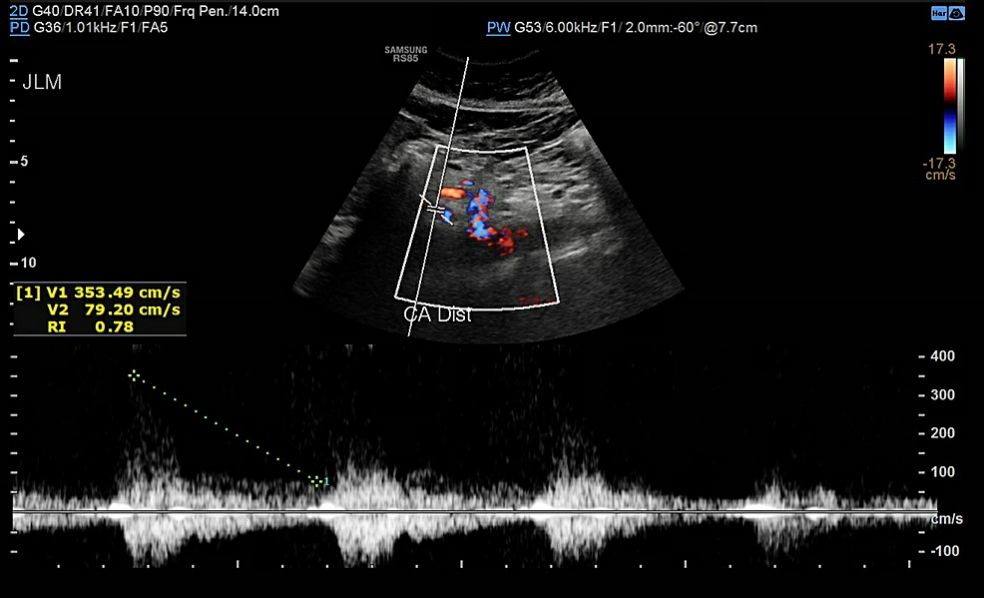
Celiac artery distally with a velocity measuring 353.49 cm/s

Subsequently, she was referred to a cardiothoracic surgeon who ordered abdominal ultrasound with a duplex that showed elevated velocities (above 200 cm/s) at various segments of the celiac artery concerning hemodynamically significant stenosis due to a compression by a prominent arcuate ligament (Figures 4-7).

 

**Figure 8:**
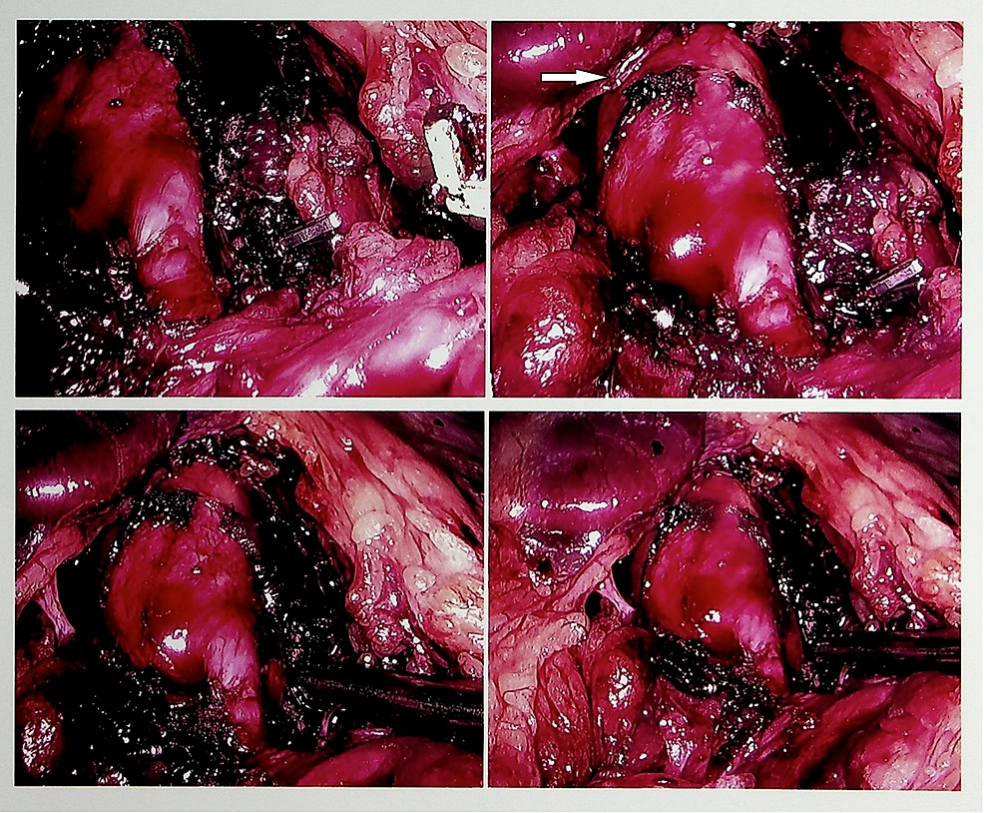
Laparoscopic removal of the median arcuate ligament (white arrow)

The findings and the underlying pathophysiology were discussed in detail with the patient who was deemed a candidate for surgical intervention. She also underwent an upper gastrointestinal endoscopy which was unremarkable. Finally, she underwent a laparoscopic procedure to divide the median arcuate ligament, which resulted in the freeing up of the celiac artery and thus in the resolution of the stenosis (Figure 8).

The patient's post-operative period was unremarkable, and she was discharged after 24 hours of the surgery. The patient has since had complete resolution of her symptoms, and she has been having regular follow up with both her primary care physician and her gastroenterologist.

## Discussion

Median arcuate ligament syndrome (MALS) is known by various names, including celiac axis compression syndrome, Dunbar syndrome, or Harjola-Marable syndrome. The syndrome is characterized by the compression of the celiac trunk by the median arcuate ligament, leading to a constellation of symptoms. The exact prevalence is unknown, as the diagnosis itself remains controversial [1]. Researchers retrospectively reviewed computer tomography angiographies (CTAs) of asymptomatic individuals undergoing live kidney donation and found that celiac artery compression >50% by the MALS occurred in 3.4% of patients [2]. Other researchers have reviewed 400 celiac angiograms taken of asymptomatic patients undergoing chemoembolization of hepatic tumors and found that 7.3% had stenosis of the celiac artery >50% in addition to having a >10 mmHg pressure gradient [3]. It has even been estimated that as many as 10-24% of the general population has celiac artery compression, but the vast majority remain asymptomatic [4].

MALS is more likely to affect women than men (4:1 ratio) and commonly presents between 30 years and 50 years of age [1]. In symptomatic patients, presenting complaints are often abdominal pain, nausea, vomiting, and weight loss. One study of 36 patients found that abdominal pain was the most common clinical feature (94%), followed by post-prandial abdominal pain (80%), nausea and vomiting (55.6%), weight loss (50%), and bloating (39%) [5]. Similarly, another study evaluated patients with unexplained chronic abdominal pain in 91%, weight loss in 40%, and nausea in 30% of patients [6]. Physical examination findings are non-specific and may include abdominal tenderness and bruits [5].

MALS symptoms closely resemble many other abdominal disorders, so it is often considered a diagnosis of exclusion. Algorithms for diagnosis have been proposed [1]. Patients with MALS often have unremarkable findings on abdominal computed tomography, upper gastrointestinal endoscopy, hepatobiliary iminodiacetic acid (HIDA) scan, and abdominal ultrasound [7]. Duplex ultrasound is the recommended initial assessment, as it is inexpensive and non-invasive [8]. For celiac artery, a peak systolic velocity of > 240 cm/s and >320 cm/s showed the highest sensitivity, specificity, and overall accuracy at >50% and >70% stenosis [9]. Additional studies include CTA and magnetic resonance angiography to provide three-dimensional visualization of the celiac compression and detect concomitant pathology [1,7]. Researchers reviewed CT scans of 23 patients with MALS and found that 16 (69.6%) had significant collateral arterial circulation while 11 (36.4%) patients had visceral artery aneurysms [10].

The underlying cause of MALS is poorly understood. It is thought to arise from anatomical variation in the origin of the MAL and celiac trunk [8]. Management of MALS involves decompression of the celiac artery and a ganglionectomy, excision of the celiac ganglion, to address neuropathic pain. Decompression was originally accomplished by an open approach with a midline laparotomy described by Dunbar et al. [11]. Roayaje et al. reported using a laparoscopic approach to release the arcuate ligament in 2000 [12]. A review of studies reporting surgical outcomes of MALS found that 85% of patients experienced immediate symptomatic relief post-operatively (laparoscopic group 96%; open group 78%) [13]. Persistent symptoms following decompression should be followed up with a CT angiography to measure pressure gradients in the celiac artery. If blood flow is still impaired or artery malformations are noted, it suggests the need for stenting or angioplasty [1]. Despite success with surgical intervention, the long-term prognosis of MALS varies. Factors associated with favorable patient outcomes included post-prandial pain, age between 40 years and 60 years, and 20 pounds or more weight loss, and unfavorable outcomes were associated with age greater than 60 years, atypical pain patterns [14]. Studies have shown a higher association of underlying psychiatric illnesses in patients of MALS and significantly lower post-operative outcomes in terms of quality of life for this cohort [15].

## Conclusions

Our case demonstrates that the clinicians need to keep in mind the diagnosis of MALS in a patient presenting with non-specific abdominal symptoms after other causes have been ruled out. The treatment is surgical with either the traditional approach or the laparoscopic decompression.
